# A Food Frequency Questionnaire for the Assessment of Calcium, Vitamin D and Vitamin K: A Pilot Validation Study

**DOI:** 10.3390/nu2080805

**Published:** 2010-07-28

**Authors:** Janet M. Pritchard, Tinasha Seechurn, Stephanie A. Atkinson

**Affiliations:** 1 Faculty of Health Sciences, McMaster University, 1200 Main St W. Hamilton ON L8N 3Z5, Canada; Email: pritcjm@mcmaster.ca (J.M.P); tinasha.seechurn@learnlink.mcmaster.ca (T.S.); 2 Department of Pediatrics, McMaster University, 1200 Main St W. Hamilton ON L8N 3Z5, Canada

**Keywords:** osteoporosis, bone, calcium, vitamin D, vitamin K, food frequency questionnaire, validation

## Abstract

The study objective was to validate a food frequency questionnaire (FFQ) to assess calcium, vitamin D and vitamin K intakes in overweight and obese postmenopausal community-dwelling women. The FFQ was validated against intakes derived from a 5-day diet record (5DDR) that also included assessment of supplement intake. Strong correlations between methods were observed for all nutrients (r = 0.63, 0.89, 0.54 for calcium, vitamin D and vitamin K, respectively) and cross-classification analyses demonstrated no major misclassification of participants into intake quartiles. Bland-Altman analysis showed that the FFQ overestimated intakes for calcium, by 576 mg/day (95% CI, −668 to 1,821 mg/day), for vitamin D by 75 IU/day (95% CI, −359 to 510 IU/day), and forvitamin K by 167 mcg/day (95% CI, −233 to 568 mcg/day). This pilot study showed promising validation evidence for the use of this FFQ, which focuses on calcium, vitamin D and vitamin K intakes in postmenopausal women, as a screening tool in clinicaland research settings.

## 1. Introduction

Postmenopausal women are at particular risk of experiencing osteoporosis-related fractures (*i.e.*, fractures of the hip, wrist, spine) [1]. Such fractures are associated with a significant increase in morbidity and mortality, and a reduction in quality of life [1,2,3]. Though the etiologies of osteoporosis and fractures are multifactorial, calcium, vitamin D and vitamin K intakes through diet and supplementation have been suggested to impact bone mineral density (BMD), fracture and fall outcomes [4,5,6,7,8,9,10]. However, as reported in a meta-analysis, evidence around the relationship between bone-nutrient intakes and BMD, fracture and fall outcomes from prospective studies and randomized controlled trials are not always consistent [11]. Studies are often limited and results heterogeneous as baseline dietary intakes of calcium, vitamin D and vitamin K are not consistently nor appropriately assessed in a study population-specific manner. 

Nutrient intakes can be estimated through the use of various tools, which differ depending on study objectives, design and resources. Typically, food frequency questionnaires (FFQ) are used in a clinical screening setting or in epidemiologic studies to assess dietary intakes, often in relation to the development of a disease [12]. A thorough review of the utilization of FFQs revealed that randomized controlled trials, cross-sectional, case-control, and cohort studies have all incorporated FFQ outcome measures (assessment of food, food groups, or nutrient intakes) into research protocols [12]. The dietary information derived from FFQs allows researchers to characterize a cohort based on nutrient intake, examine the relationships between diet and disease, and diet and other study outcome measures, such as biochemical and functional measures [12,13,14]. Regardless of the setting or purpose, the questionnaire should be validated and compared to a gold standard diet analysis technique for the specific population under study. In particular, it should be country-specific, age-specific and include a comprehensive list of food items to capture the study population’s eating patterns, food choices and diet variability [12,15,16]. 

Previously, an FFQ was developed to estimate a broad range of nutrient intakes in a multicultural cohort of women living in Southern Ontario, Canada [17]. This FFQ consisted of foods derived from the Canadian Study of Diet, Lifestyle and Health FFQ food list, and FFQ food-lists specific to individuals of South Asian, Chinese and European descent living in Canada [17,18]. The FFQ employed in this study was adapted in style, but revised by focusing on only foods containing significant amounts of calcium, vitamins D and K from the FFQ that was previously validated, although not for vitamins D and K [17]. Also, at the time of the initial validation, calcium fortified foods were not included as they did not exist on the market. Therefore, the objectives of the present study were to: 1) update the original FFQ with a focus on including an assortment of food items containing bone-related nutrients; 2) use the improved FFQ in a cohort of postmenopausal women; 3) validate the use of the FFQ to estimate the average intakes of calcium, vitamin D and vitamin K over a year in postmenopausal women.

## 2. Experimental Section

### 2.1. Participant Recruitment

Individuals who were participating in a larger primary study were approached to participate in the FFQ validation study. This larger study has been registered at Clinicaltrials.gov and holds the identifier, NCT00982371. A convenience sample of 15 community-dwelling postmenopausal women was derived from the larger sample, consisting of 65 participants who resided in Hamilton and surrounding area in Ontario, Canada. Participant recruitment occurred in-person during a study visit for the larger study, and over the telephone from January-February 2009. For inclusion into the larger study, participants must have met the following inclusion criteria: 1) postmenopausal for more than 5 years (menopause was defined as 12 months after the cessation of normal menstrual cycles, in accordance with the World Health Organization definition); and 2) greater than or equal to 65 years of age. Exclusion criteria for the primary study were: 1) use of medication in the previous 24 months known to affect bone, such as hormone therapy, calcitonin, selective estrogen receptor modulator, fluoride, parathyroid hormone, or bisphosphonate; 2) systemic glucocorticoid use for more than 3 months at a dose of more than 2.5mg/day; 3) history of metastatic cancer (*i.e.*, breast) in the past 5 years; 4) diagnosis of intrinsic bone disease (*i.e.*, Paget’s disease, Cushing’s Syndrome); 5) untreated malabsorption syndrome (*i.e.*, Celiac disease); 6) self-reported hyperparathyroidism or hypoparathyroidism; 7) severe renal impairment (Cockcroft-Gault glomerular filtration rate <30 mL/min). For the FFQ validation study, participants had to be able to record their food and beverage intake for five non-consecutive days (3 weekdays, 2 weekend days). In addition, participants completed a questionnaire on current medication use (including dietary supplements), living arrangements, ambulation status and diagnoses of chronic diseases. All participants signed informed consent documents. The McMaster University Faculty of Health Sciences/Hamilton Health Sciences Research Ethics Board reviewed and approved this study. 

### 2.2. Nutrient Analysis

#### 2.2.1. Food Frequency Questionnaire (FFQ)

The interviewer-administered FFQ contained 161 food items that are found to contain ≥30 mg of calcium, ≥10 IU of vitamin D3, or ≥1 mcg of vitamin K per average serving size. The foods are arranged into 9 categories based on food grouping (*i.e.*, “dairy/egg products”, “fruits” *etc.*). For each food item, participants were asked if the food item is normally consumed at least once a month, and if so, how often it is consumed (*i.e.*, frequency per day, per week, or per month). The participants were then asked in what quantity the food-item is consumed (*i.e.*, smaller than average size listed, average size, or larger than the average size listed). The list of foods included specific items for calcium/vitamin D-fortified orange juice, cow’s milk and soy beverage. A photograph album was used as a participant aid while completing the FFQ to assist participants in identifying the food in question and serving sizes. For assessment of nutritional supplements, specific sections of the FFQ were used to record vitamin/supplement combinations, including calcium supplements (with seven categories of amounts), calcium with vitamin D supplements (with eight categories of amounts), vitamin D supplements (with six categories of amounts), vitamin K supplements (with eight categories of amounts), and a section for “other health or nutritional products” not included in the above (e.g., calcium-fortified water).

As decided on *a priori, *only complete FFQs were analyzed. Nutrient intakes were computed using an in-house FFQ calculator (Microsoft Office Excel 2003, USA). This FFQ calculator is based on the participant’s frequency of consumption, amount of the item consumed (calculated as 0.5 for smaller than, and 1.5 for larger than average serving size) and amount of nutrient in the serving size indicated. Nutrient values for each food item were derived from the 2008 United States Department of Agriculture (USDA) National Nutrient Database for Standard Reference, the 2007 Health Canada Canadian Nutrient File (CNF), and from a previously developed FFQ nutrient calculator [17,19,20]. The USDA National Nutrient Database for Standard Reference is the standard reference database reporting the amount of nutrients in over 7,500 foods commonly consumed in the United States. The CNF is the standard reference database reporting the amount of nutrients in over 5,500 foods commonly consumed in Canada. These two open-access databases were used for nutrient analysis because of the wide variety of frequently updated foods included in each. In addition, the CNF was used to obtain nutrient information on various bread and cereal products, vegetable oils, margarine, and dairy products because calcium and vitamin D fortification practices in the USA and Canada differ [21,22]. This ensured that the FFQ in-house calculator contained up-to-date nutrient values for the food items. 

#### 2.2.2. 5-Day Diet Record (5DDR)

The 5DDR was selected as the reference method for this validation study because we were interested in capturing the participant’s habitual eating patterns over 2 weekend days and 3 weekday days, and to ensure that vitamin K intake was captured [21,22]. Participants were also requested to indicate how the food item was prepared to provide a more accurate estimation of nutrient intake (*i.e.*, pan fry with vegetable oil, versus boil with water). The 5DDR was completed by all participants within one month following the administration of the FFQ. Participants indicated if they took a calcium, vitamin D, vitamin K or multivitamin supplement, consistent with the FFQ assessment. Of note, the 5DDRs were not reviewed with the participants upon completion and submission to the research assistant (TS). Using data from the supplement manufacturer’s website or product labels, the amount of calcium, vitamin D and vitamin K from the supplement was added to the participant’s intake. Nutrient intakes were calculated using diet analysis software (Nutritionist Pro, Axxya Systems, Stafford, Texas USA), which is also based on USDA National Nutrient Database for Standard Reference and Health Canada’s CNF version 2007b [20,23,24]. All nutrient analysis procedures were conducted by a single investigator (TS).

### 2.3. Statistical Analysis

The mean ± SD intakes for each nutrient (calcium, vitamin D, and vitamin K) were calculated. Frequency statistics were computed for the additional descriptive characteristics. The mean intake values derived from the FFQ and 5DDR were compared using a paired 2-tailed Student t-test. 

To demonstrate robustness of the validation technique, several statistical methods were utilized. To determine whether the intakes derived from the FFQ were related to the intakes derived from the 5DDR, Pearson correlation coefficients were used. The Bland-Altman method was used to assess the agreement between FFQ and 5DDR across a range of nutrient intakes. A cross-classification analysis was used to determine whether the FFQ and 5DDR have good agreement, or misclassify participants into categories based on intake levels. In order to perform the cross-classification analysis, the intakes for calcium, vitamin D and vitamin K derived from the FFQ and 5DDR were divided into quartiles. The proportions of participants were computed who were classified into the same quartile, the same ±1 quartile, or who were entirely misclassified after FFQ and 5DDR assessment. To identify those most at risk of inadequate nutrient intake, the dietary reference intake (DRI) for each nutrient was used as an intake cut-off. The following cut-off points were used: for calcium, the adequate intake (AI) of 1,200 mg/day; for vitamin D, the AI of 600 IU/day; and for vitamin K, the AI level of 90 mcg/day [19]. Each subject was classified as having nutrient intake above or below the corresponding AI. The FFQ sensitivity was defined as the proportion of participants with intake levels below the AI and the specificity was defined as the proportion of participants with intake levels above the AI according to results from the FFQ and 5DDR.

Statistical analyses were performed with SPSS (Statistical Package for the Social Sciences) version 15.0 for Windows (SPSS Inc., Chicago, Illinois) and MedCalc Software (Version 10.3, Belgium). A p-value of <0.05 was considered significant for this study.

## 3. Results and Discussion

Of the 25 women approached to participate in this study, 5 women declined participation, and 5 women failed to complete and return the 5DDR. Therefore, the results presented here reflect data from 15 women who completed the FFQ and 5DDR. Table 1 displays the demographic characteristics of the study participants, whose mean age was 70.3 ± 4.7 years. Calcium and vitamin K intakes derived from the FFQ and 5DDR were significantly different (p < 0.05), but not for vitamin D intakes (Table 2). However, nutrient intakes derived from the FFQ and 5DDR were positively correlated, with the strongest correlation between methods for vitamin D intake (Table 2). 

**Table 1:** Descriptive characteristics of participants (N = 15).

	Proportion, n (%)
**Ethnicity**	
North-American Caucasian	11 (73.3)
European	2 (13.3)
South American	1 (6.7)
Southeast Asian	1 (6.7)
**Ambulation status**	
No aid	11 (73.3)
Walking aid	4 (26.7)
**Living arrangements**	
Living independently	3 (20.0)
Living with family support	9 (60.0)
Living independently with non-live in support	3 (20.0)
**Number of years since menopause**	
11–15 years	3 (20.0)
16–20 years	2 (13.3)
>20 years	10 (66.7)
**Body mass index classification**	
Normal weight (18.5–24.99 kg/ m^2^)	0
Overweight (25–29.99 kg/ m^2^)	4 (26.7)
Obese (≥30 kg/ m^2^)	11 (73.3)
**Self-reported diagnosis of chronic disease**	
Osteoporosis	3 (20.0)
Osteoarthritis	5 (33.3)
Type 2 diabetes	12 (80.0)
**Number of prescription medications**	
None	1 (6.7)
1–5 medications	4 (26.7)
6–10 medications	4 (26.7)
11–15 medications	1 (6.7)
16–20 medications	4 (26.7)

**Table 2:** Nutrient intakes (mean ± SD) derived from FFQ and 5DDR, and correlation between methods.

	161 item FFQ			5DDR			*p-value*	*Pearson r*
	Diet sources	Supplement sources	Total	Diet sources	Supplement sources	Total		
**Calcium** (mg/day)	1,191 ± 671	640 ± 551	1,831 ± 788	615 ± 292	640 ± 551	1,255 ± 492*	0.003	0.63‡
**Vitamin D **(IU/day)	226 ± 156	708 ± 494	934 ± 464	151 ± 209	708 ± 494	859 ± 485	0.253	0.89‡
**Vitamin K** (mcg/day)	266 ± 77	21 ± 51	287 ± 228	99 ± 77	21 ± 51	120 ± 84*	0.009	0.54‡

*Indicates significant difference between FFQ and 5DDR intakes, p < 0.05. Significant difference between dietary sources determined by independent samples 2-tailed t-test. ‡ Indicates significant correlation between FFQ and 5DDR, p < 0.05

**Figure 1:**
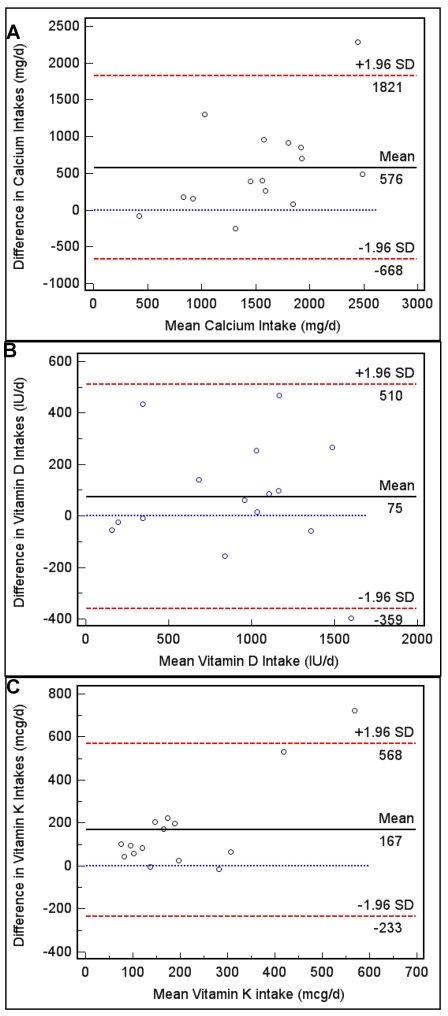
(A) Bland-Altman plots to assess agreement and systematic difference between the FFQ and 5DDR for calcium intake, (B) vitamin D intake and, (C) vitamin K intake.

**Table 3:** Cross-classification analysis to determine proportion of participants classified into the same, or same ± 1 quartile based on FFQ and 5DDR intakes.

	% classified intosame quartile	% classified into same ± 1 quartile	% misclassified
**Calcium**	47	87	0
**Vitamin D**	73	100	0
**Vitamin K**	33	87	0

For calcium: quartiles 1 to 4 for FFQ were <1,185, 1,185–1,764, 1,765–2,262, >2,262 mg/d; Quartiles 1 to 4 for 5DDR were <841, 841–1,348, 1,349–1,461, >1,461 mg/d; For vitamin D: quartiles 1 to 4 for FFQ were <561, 561–1,040, 1,041–1,210, >1,210 IU/d; quartiles 1 to 4 for 5DDR were <346, 346–925, 926–1,060, >1,060 IU/d; For vitamin K: quartiles 1 to 4 for FFQ were <135, 135–248, 249–286, >286 mcg/d**; **quartiles 1 to 4 for 5DDR were <59, 59–79, 80–150, >150 mcg/d.

## 4. Discussion and Conclusions

A valid, comprehensive tool to assess the intakes of key bone-nutrients is essential in skeletal health research involving humans, such as randomized controlled trials, cohort and case-control studies [25,26]. In the present study, we demonstrated that a previously developed 161-item FFQ is valid for the assessment of calcium, vitamin D and vitamin K intakes in a cohort of overweight and obese community-dwelling postmenopausal women. In general, the calcium, vitamin D and vitamin K intakes derived from the FFQ were significantly related to the intakes derived from the 5DDR. Furthermore, when a participant’s intake was classified into one of four quartiles for each method of assessment, there were no instances where a participant was misclassified. This FFQ appears to perform well in a cohort of postmenopausal women, however, it appears to perform best when the intakes are similar to the adequate intake levels, particularly for calcium and vitamin K [12].

In addition to the FFQ, dietary assessment can be performed using various other tools, which are associated with their own strengths and weaknesses. While the weighed food record performs well against an FFQ because portions are weighed prior to consumption, this method may interfere with an individual’s daily life and habitual dietary intake [12]. The 24-hour recall method is often preferred over the weighed food record because it is less demanding on a study participant, but tends to rely more heavily on memory and the ability to conceptualize portion sizes [12]. From a logistical perspective, an FFQ is an attractive dietary assessment tool for use in health research and some clinical settings due to the low cost of administration and processing, and low respondent burden. The additional strengths of this FFQ were its ease of administration, detailed food list, and consideration of seasonal foods. In southern Ontario, Canada, the consumption of food items varies as the seasons change from winter to summer. Therefore, by including foods on the FFQ that are consumed on a seasonal basis (*i.e.*, consumed primarily in the spring and summer, such as broccoli) the opportunity to capture the participant’s average dietary pattern over several months is improved.

Ease of administration of this FFQ was enhanced by the use of a food photograph album that emphasized portion sizes. This aid contributed to the interviewer’s ability to complete this FFQ with the participant in under 25 minutes, which seems to be the average time appropriate for FFQ administration [12]. This FFQ contained 161 food items including specific items for calcium and vitamin D fortified foods, which made the food list longer than other FFQ food lists [24,27]. The benefits of using an FFQ with a greater number of food items are improved accuracy in food intake estimates, and improved ability to capture the variability in the population’s diet. A previous study attempted to shorten an FFQ by reducing the number of food items, however, an adjustment factor had to be applied to the calcium intake derived from the FFQ due to nutrient intake underestimation [12]. 

Since it was established that nutrient intake has an effect on bone health, and should be considered in epidemiological study analyses, researchers have validated FFQs for the assessment of calcium, vitamin D, vitamin K and other macronutrients [27]. However, ours is the first FFQ to be validated for the assessment of 3 prominent bone nutrients: calcium; vitamin D; and vitamin K. As noted by Serra-Majem and colleagues, as the number of validated FFQs increases, certain criteria should be applied to assess the quality of the validation study. Specifically, the sample and sample size, statistical analysis, mode of data collection, inclusion of seasonal foods and supplements should be considered [24,27,28,29,30,31,32,33,34,35]. 

Previously, calcium FFQs have been validated for use in Italian [36], Malaysian [33], Brazilian [29], Vietnamese [35] and general American cohorts [31]. Validating the FFQ for the specific population under study is essential for improving apparent validity. As age and cognitive function do not have a significant impact on FFQ validity [27,30,34], we validated the FFQ in older postmenopausal women, where as others have validated the use of calcium and vitamin D specific FFQs in younger populations [37,38]. Our study sample was made up of 15 participants, which is a study limitation. The suggested sample size for an FFQ validation study is 50–100 individuals [27,30,31,34], however, others have used fewer study participants [12,36] and similar to the present study, produced promising results. While small in number, our study sample consisted of individuals with varying levels of independence, as reflected by ambulation status and living arrangements, and chronic disease diagnoses, which improves the external validity and generalizability of the FFQ to be used in other study samples consisting of postmenopausal women. It should be noted however that all study participants were overweight or obese. 

The FFQ in the present study overestimated nutrient intakes when compared to the reference method, which resulted in an overall questionnaire systematic bias, consistent with previous reports [27,35]. This overestimation could be attributed to: 1) participant awareness of the present study’s objective to assess bone-related nutrient intakes or the larger study’s objective to assess bone health; 2) the number of food items in the FFQ [34,35]; 3) or the lack of multiple 5DDR assessments throughout the year producing an underestimation of intake derived from the reference method [39]. The systematic bias for calcium and vitamin K was more evident for higher intake levels, and less evident for intake levels around the AI. This suggests that this FFQ would perform the best in populations where the intake levels were approximately at the AI levels. We speculate that this FFQ would not perform as well in populations where the intake levels derived from the FFQ were at, or above the DRI upper limit (UL) levels. The systematic bias for vitamin D was broader, and did not depend on the proximity to the AI, as seen with calcium and vitamin K, however, the mean difference between the intakes derived from each method was the smallest for vitamin D (difference of 75 IU/d). In order to reduce systematic bias, future FFQ validation studies should focus on the use of a questionnaire, which includes food items aligned with cultural, socio-economic, and geographic dietary trends, attempt to keep the participant blinded to the study objectives, and use more than one reference method data collection time point, if seasonal foods are included in the FFQ food list. 

Intakes derived from the FFQ were significantly correlated with intakes derived from the 5DDR. For calcium the correlation strength was similar to previous that observed in other validation studies [12] and within the range (0.50–0.70) that is considered suitable for FFQ validation studies [31,34,35]. The Bland-Altman method of analysis should be used in conjunction with correlation statistics [40]. We demonstrated that agreement between methods was fairly good for calcium, vitamin D and vitamin K, with only one (1/15) outlier observation occurring outside the 95% agreement range for each nutrient. Systematic bias did exist, and the FFQ tended to overestimate nutrient intake, however, it seemed that for calcium and vitamin K, the agreement between methods was stronger for intakes at approximately the AI levels. Similar to our findings, Sebring and colleagues also found a systematic bias when validating a Calcium Questionnaire against 7-day diet records [12]. 

Finally, we demonstrated that the FFQ was able to place all participants into the same or adjacent (same ± 1 quartile) quartile of intake for vitamin D intake, and was able to place 87% of participants into the same or adjacent quartile of intake for calcium and vitamin K. No study participants were grossly misclassified, which provides evidence that the FFQ performed well when allocating participants according to dietary intake distribution. These findings are in accordance with the findings from another validation study in postmenopausal women where approximately 50% of participants were classified into the same calcium intake quartile with a 60-item FFQ and 3-day diet records [27]. 

Additional strengths of the present validation study include: 1) the nature of data collection (*i.e.*, interviewer-administered FFQ); 2) the inclusion of a vast array of seasonal foods containing calcium and vitamin D and vitamin K and calcium/vitamin D-fortified foods; 3) inclusion of details of supplement intakes of calcium and vitamins D and K; 4) the reference method chosen. The use of the 5DDR (3 weekdays, 2 weekend days) over the 24-hour recall method is preferred in elderly study populations because of the 24-hour recall method’s reliance on memory, and problems with conceptualization of portion sizes, which may distort dietary intake [35]. On average, 4 to 5-day diet records are appropriate and cost-effective for use as a reference method in FFQ validation studies [41].

This study is not without limitations. First, the generalizability of these findings to the general population of postmenopausal females and utilisation of the FFQ for future studies must be considered. The sample size for this study was small, which limits the conclusions we can make and the generalizability of our findings. This study however provides novel pilot data for future validation studies in Canadian postmenopausal women, or other cohorts (*i.e.*, older men). Also, the larger study from which the convenience sample for the present study was drawn excluded individuals who were on osteoporosis-related therapy, therefore no participants were taking osteoporosis medication. As previously reported in a large population-based study, approximately 3% and 25% of Canadian women are on bisphosphonate treatment or hormone therapy, respectively [42]. 

Furthermore, all participants enrolled in the present study were classified as overweight or obese. It is possible that the narrow range in BMI measurements of our study participants may influence the generalizability of our data, as studies have demonstrated that obese women have significantly lower calcium intakes, compared to non-obese women of the same age, and that lower calcium intake is associated with insulin resistance, the hallmark of type 2 diabetes [43,44]. It has been reported however that overweight and obese individuals tend to underreport food consumption in dietary assessment studies, which may contribute to the relationship between low calcium intake and obesity [45,46]. Of note, when compared to total intake levels (dietary and supplementary sources) of Canadian women over the age of 71 years derived from the 2004 Canadian Community Health Survey (CCHS) which is based on a 24-hour recall assessment, the present study cohort had similar 5DDR intake levels of calcium (1,255 ± 492 mg/day, 5DDR *vs.* 1,638 mg/day, CHHS) [47]. When Vatanparast and colleagues assessed vitamin D dietary intake levels using the CCHS data, dietary levels from food sources alone were lower than reported in the present study (859 ± 485 IU/day, 5DDR vs. 244 ± 28 IU/day, CCHS) for all Canadian women over 71 years, however this study excluded intake levels from supplements [48]. In urban Caucasian Canadian women over 51 years, the average dietary and supplementary intake of vitamin D was reported to be 544 ± 460 IU/day, which is comparable to our 5DDR vitamin D estimate [13]. The vitamin K levels reported in this study (120 ± 84 mcg/day) were also similar to those assessed by a 5DDR in 30 elderly Canadian women and 9 elderly Canadian men, where the mean intake level was 135 ± 154 mcg/day [24].

With respect to the study methodology, the participants were aware of the study objectives, which may have influenced FFQ responses and 5DDR entries, distorting the habitual intakes of calcium, vitamin D and vitamin K. Also, only one reference method was used and participants were asked to complete the 5DDR only once. It has been suggested by others that multiple reference methods, including dietary methods and biochemical analyses, be used in validation studies [12,36]. If the FFQ assesses consumption of nutrients over the year (*i.e.*, spanning 4 seasons), multiple time point collections for the reference method should occur [1]. However, the majority of previous FFQ validation studies only included one reference method in the analysis [12,36]. Finally, to ensure that the FFQ is robust for use by different investigators or clinical staff, the inter-rater and intra-rater reliability should be assessed. This assessment was beyond the scope of the present study, but should be considered in future FFQ validation studies. 

In conclusion, the present study provides promising pilot validation evidence for the use of a “bone health” FFQ that focuses on calcium, vitamin D and vitamin K in postmenopausal women. Though the FFQ is not a perfect dietary assessment tool, it can classify individuals into the same or adjacent quartile of calcium, vitamin D and vitamin K intakes. The FFQ proved to be a sensitive and specific tool for classifying individuals into calcium and vitamin D adequate intake categories defined by the Institute of Medicine’s DRI recommendations [27,30,31,34,35]. These findings make this FFQ particularly attractive for use in a clinical screening setting for nutrient deficiency where resources may be limited, in a study eligibility screening environment, and in skeletal research involving postmenopausal women. Future research should aim at validating this FFQ in a larger study population, and validating this FFQ for use in men and other clinical populations at high risk for fracture.
